# Recovery of SIRT3-SOD2 Axis and Mitophagy by Short-Term Calorie Restriction in Old Rat Soleus Skeletal Muscle

**DOI:** 10.3390/antiox14091125

**Published:** 2025-09-17

**Authors:** Rosa Di Lorenzo, Anna Picca, Guglielmina Chimienti, Christiaan Leeuwenburgh, Vito Pesce, Angela Maria Serena Lezza

**Affiliations:** 1 Department of Biosciences Biotechnologies and Environment, University of Bari Aldo Moro, Via Orabona 4, 70125 Bari, Italy; rosa.dilorenzo@uniba.it (R.D.L.); guglielminaalessandra.chimienti@uniba.it (G.C.); 2 Department of Medicine and Surgery, LUM University, 70100 Casamassima, Italy; picca@lum.it; 3 Fondazione Policlinico Universitario “A. Gemelli” IRCCS, 00168 Roma, Italy; 4 Department of Aging and Geriatric Research, Institute on Aging, Division of Biology of Aging, University of Florida, Gainesville, FL 32611, USA; cleeuwen@ufl.edu

**Keywords:** resveratrol, rat soleus skeletal muscle, caloric restriction, mitochondrial biogenesis, mitophagy, aging

## Abstract

Age-related mitochondrial dysfunction is involved in the progressive loss of mass and strength of skeletal muscle with aging. The effects of a short-term calorie restriction (ST-CR) were assessed in the oxidative skeletal soleus muscle (Sol) from 27-month-old rats and compared with those of a CR in combination with resveratrol (RSV) (ST-CR + RSV). PGC-1α and PRXIII proteins showed a marked decrease in both ST-CR and ST-CR + RSV rats. The SIRT3 protein presented a very relevant increase in both ST groups. ST-CR and ST-CR + RSV elicited a marked increase in SOD2 protein amount and activity. ST-CR and ST-CR + RSV led to recovery of the SIRT3-SOD2 axis as a fast/early response. ST-CR and ST-CR + RSV did not affect the MFN2 protein, whereas both treatments induced a relevant increase in DRP1 protein. ST-CR and ST-CR + RSV induced a decrease in Parkin protein, suggestive of rescued mitophagy, leading to the elimination of dysfunctional mitochondria. Such a response likely enhanced the fission-mediated elimination of mitochondria, supported by the marked increase in DRP1. MtDNA copy number and TFAM protein were not changed by any ST treatment. The mtDNA oxidative damage level was strongly increased by both ST treatments. All the effects elicited by ST-CR and ST-CR + RSV were specific to the oxidative type fibers.

## 1. Introduction

**Figure 1:**
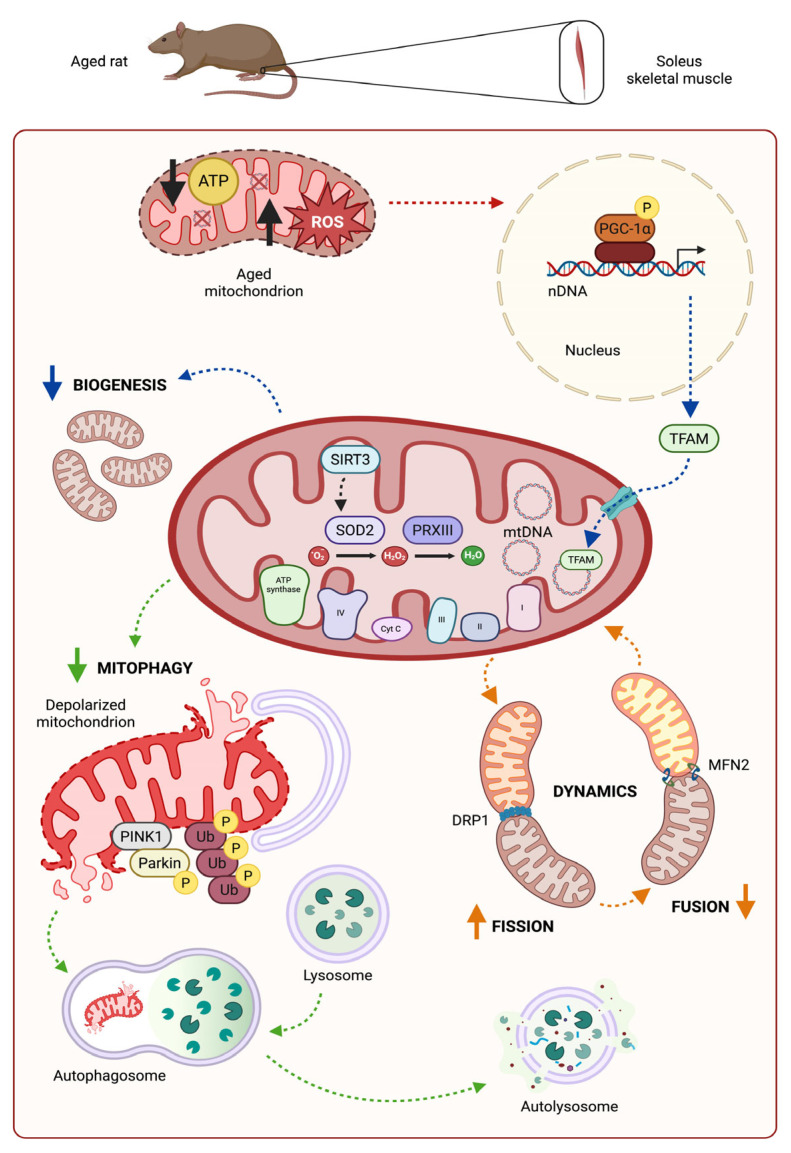
Schematic diagram of relevant mitochondrial pathways in aged rat soleus skeletal muscle. Aged rat soleus skeletal muscle harbors dysfunctional mitochondria deficient in ATP content and presenting an oxidative stress condition featuring abundant ROS, which are neutralized by antioxidant enzymes such as SOD2, induced by SIRT3, and PRXIII. These aged organelles exhibit reduced biogenesis, normally involving PGC-1a and TFAM; decreased PINK1/PARKIN-mediated mitophagy; and unbalanced dynamics, including fission proteins (i.e., DRP1) and fusion proteins (i.e., MFN2). Dashed arrows indicate functional processes. Bold arrows mean decrease or increase of the respective process/molecular species. Abbreviations: I, II, III and IV, mitochondrial respiratory complexes I through IV; ATP, adenosine triphosphate; Cyt C, cytochrome C; DRP1, dynamin-1-like protein; MFN2, mitofusin 2; mtDNA, mitochondrial DNA; nDNA, nuclear DNA; P, phosphate group; PGC-1α, peroxisome proliferator-activated receptor gamma coactivator-1α; PINK1, PTEN-induced kinase 1; PRXIII, peroxiredoxin III; ROS, reactive oxygen species; SIRT3, sirtuin 3; SOD2, manganese superoxide dismutase 2; TFAM, mitochondrial transcription factor A; Ub, ubiquitin. Created in Biorender. Pesce, V. (2025) https://app.biorender.com/illustrations/68af02639d99ad184203a54b?slideId=4d9da8ad-8cd0-40f7-a425-341fbf705dff (accessed on 4 September 2025).

Aging is a complex natural process marked by a structural and functional progressive decline of an organism’s systems [1]. Extensive work has been carried out to list the hallmarks of aging [2] and to interpret their significance [3,4]. All lists of hallmarks of aging include mitochondrial dysfunction because the functional deterioration of the organelles deeply impacts cell metabolism and fate. Mitochondrial dysfunction, as the whole process of aging, features a marked tissue-specificity [5]. Aging affects skeletal muscle through the progressive loss of mass (atrophy) or mass and strength (sarcopenia), and the age-related increase in muscle fatigability appears to also be due to the accumulation of dysfunctional mitochondria, impairing muscle oxidative and endurance capacity [6]. Dysfunctional organelles might accumulate because of age-related impairments in pathways cooperating to maintain mitochondrial health, such as mitochondrial dynamics (resulting from the balance between fusion and fission) and mitophagy (the process for removal of dysfunctional mitochondria) [7], as well as mitochondrial biogenesis and sirtuin pathways [8,9] (Figure 1). Several interventions have been tested for their potential to delay aging, and calorie restriction without malnutrition (CR) has been shown to be the most robust nongenetic and nonpharmacological approach efficient in warding off the cellular markers of aging [10]. CR induces a large number of physiological adaptations, some of which are tissue-specific [11]. Amelioration of mitochondrial function is one of the common CR traits [12], although it induces different effects in mitochondrial morphology and dynamics depending on fiber [13] or muscle type (i.e., oxidative soleus vs. glycolytic white gastrocnemius) [14]. CR was shown to decrease mitochondrial reactive oxygen species (ROS) production [15,16] and to reduce the age-related mitochondrial oxidative stress, also through the presence of more efficient organelles. Therefore, it is widely accepted that CR stimulates mitochondrial biogenesis, likely by way of the activation of mediators, such as the master regulator peroxisome proliferator-activated receptor gamma coactivator-1α (PGC-1α) [17]. However, the CR-mediated decrease in oxidative stress might also be achieved through the increase in mitochondrial antioxidant proteins, which can be exemplified by peroxiredoxin III (PRXIII) [18] and manganese superoxide dismutase 2 (SOD2) [19]. Furthermore, the intramitochondrial balance between activity of ROS-mediated signaling and neutralization of damaging ROS is obtained by means of the cooperation/interplay of various pathways, such as that of sirtuins [20], with a relevant role played by sirtuin 3 (SIRT3) [9]. In particular, SIRT3 can directly induce clearance of ROS by deacetylation and activation of SOD2 [21], and this was one of the first demonstrations of the SIRT3-SOD2 axis, which was further found in a growing number of different experimental situations [22]. SIRT3 has a pleiotropic function in mitochondria, which also includes its crucial regulatory activity of mitochondrial dynamics, deeply involved in the mitochondrial quality control mechanisms [23]. Last but not least, SIRT3 has been shown to be involved in the regulation of mitophagy [24], and mitophagy impairment has been increasingly indicated as a feature of the age-related mitochondrial dysfunction [25]. The assessment of mitophagy can be carried out through the analysis of two proteins very relevant for the process, namely PTEN-induced kinase 1 (PINK1) and E3 ubiquitin–protein ligase Parkin (Parkin) [26]. To draw a detailed picture of mechanisms contributing to the beneficial actions of CR on mitochondrial dysfunction, also the pathways involved in mitochondrial DNA (mtDNA) maintenance, which imply regulation of mtDNA copy number and level of oxidative damage and expression of the histone-like protein mitochondrial transcription factor A (TFAM) have to be analyzed. Overall, cell mitochondrial abundance is regulated through the complex interplay of mitochondrial quality control pathways, namely biogenesis, dynamics, and mitophagy [27], which are all affected by CR. Furthermore, the literature has clearly shown that CR outcomes may depend on the age of the animal at the beginning of the nutritional treatment (e.g., young vs. middle or old age) [14,28]. Also, the duration of the diet, namely whether it is administered for a short-term (ST, one–two months) [28] or for a long-term (LT, 13–20 months) [13,14,29,30], may affect the results. However, the experimental practice of CR is unlikely to be applied in humans because of its inherent burdensome compliance, and such difficulty has fostered the search for agents that may mimic some outcomes of CR in subjects fed a normal caloric intake. The search for CR mimetics (CRMs) focused on resveratrol (3,5,4′-trihydroxystilbene, RSV), which is probably the most widely studied compound mimicking CR [31]. Supplementation of resveratrol (RSV) has been shown to be efficacious in counteracting aging, although with tissue-specific effects [32]. For instance, the same kind of RSV treatment has been reported to exert different effects on mitochondria depending on muscle fiber type (i.e., oxidative soleus (Sol) vs. glycolytic white gastrocnemius) [33,34]. Today, it is still unclear whether the benefits of CR or RSV treatment occur via common or similar or independent pathways and the comparison between CR and combined CR + RSV (present results) and results from the previous study on RSV supplementation [33] might help to ascertain the presence of additional/cumulative or opposite/counteractive effects elicited by the alternate nutritional treatments. This represents one of the novelties in the present study, together with the short time (ST, six weeks) of administration to aged (27-month-old) rats, which were examined for the oxidative skeletal soleus muscle. According to recent reports from rodent skeletal muscle studies [14], it is necessary to assess the differential fiber type-specific responses induced by nutritional treatments to deepen the knowledge of the respective molecular mechanisms. Furthermore, the late-life initiation and the short duration of the treatments should help to verify if the positive effects, usually elicited by LT regimens, might also be induced by a short treatment begun late in life, which might increase its compliance in human subjects.

Therefore, in the present study, we analyzed the effects of ST-CR and ST-CR + RSV treatments on the protein amounts of PGC-1α, PRXIII, SIRT3, mitofusin2 (MFN2), dynamin-related protein 1 (DRP1), PINK1, and Parkin; on the expression and activity of SOD2; and on the mtDNA copy number and level of oxidative damage together with the protein amount of mitochondrial transcription factor A (TFAM).

## 2. Materials and Methods

### 2.1. Animals

In this study, 27-month-old male hybrid Fischer 344 × brown Norway rats provided by the National Institute on Aging were used following the same experimental procedure described in [34]. All experimental procedures were performed at the Department of Aging and Geriatric Research, Division of Biology of Aging, University of Florida, Gainesville, FL 32611, USA. They were approved by the Institutional Animal Care and Use Committee at the University of Florida (Study#:200902992) and performed in accordance with the National Institutes of Health guidelines. Each animal was individually kept in a cage, acclimatized for 4 weeks and then randomly assigned to one of three treatment groups: ad libitum (AL), caloric-restricted (CR) (20% reduced AL), and caloric-restricted plus resveratrol administration (CR + RSV) (20% CR and 50 mg/kg/day RSV contained in tablet with a bacon flavor). After the 6 weeks of treatment, rats were sacrificed and soleus (Sol) muscle was isolated, weighed, frozen in liquid nitrogen, and stored at −80 °C pending further analysis.

### 2.2. Western Blot Analysis

A total of 50 mg of frozen Sol was homogenized in lysis buffer and centrifuged at 13,000× *g* for 25 min at 4 °C to obtain the supernatant. Total protein content was measured using the Pierce™ BCA Protein Assay Kit (Thermo Fisher Scientific, Waltham, MA, USA). A total of 30 µg of proteins was loaded on 4–15% Criterion^TM^ TGX Stain-Free^TM^ Precast Gels (#5678085, Bio-Rad Laboratories, Inc., Hercules, CA, USA) to perform a separation by sodium dodecyl sulphate–polyacrylamide gel electrophoresis (SDS-PAGE), followed by transfer to a polyvinylidene difluoride membrane (GE10600023 Amersham™ Hybond^®^ P Western blotting membranes, PVDF; Merck KGaA, Darmstadt, Germany). Blots were blocked in TBS1X-0.1%Tween-20 containing 5% bovine serum albumin (BSA) for 1 h and subsequently incubated with different primary antibodies overnight at 4 °C: anti-PGC-1α (Novus Biologicals, Minneapolis, MN, USA, #NBP1-04676PCP; dilution 1:5000), anti-peroxiredoxin 3, PRXIII (Ab Frontier, Seoul, Republic of Korea, #LFPA0030; 1:10,000), anti-SIRT3 (Sigma-Aldrich Merck, KGaA, Darmstadt, Germany, #SAB5700222; 1:10,000), anti-SOD2 (MnSOD, Cell Signaling Technology Inc., Danvers, MA, USA #13194S; dilution 1:10,000), anti-MFN2 (Cell Signaling Technology Inc., Danvers, MA, USA #9482S; dilution 1:10,000), anti-DRP1 (Abnova, Taipei City, Taiwan, #H00010059-M01; dilution 1:20,000), anti-PINK1 (Proteintech, Rosemont, IL, USA #23274-1-AP; dilution 1:5000), anti-Parkin (Proteintech, Rosemont, IL, USA #14060-1-AP; dilution 1:5000), anti-TFAM (Cell Signaling Technology Inc., Danvers, MA, USA #7495S; dilution 1:10,000), and anti-β-actin (Sigma-Aldrich Merck, KGaA, Darmstadt, Germany, #A5316; dilution 1:10,000). The following day, after three 10 min washes with TBS1X-0.1% Tween-20, membranes were incubated with a secondary antibody (Cell Signaling Technology Inc., Danvers, MA, USA, anti-rabbit #7074; anti-mouse #7076), combined with horseradish peroxidase (dilution 1:10,000) for 1 h, and revealed with ECL detection through the ChemiDoc System. For the molecular weight estimation, the marker Precision Plus Protein Dual Color Standards (Bio-Rad Laboratories Inc., Hercules, CA, USA #1610374) was used. Stripping, performed with Restore Stripping Buffer (ThermoFisher Scientific, Waltham, MA, USA #21059), and reprobing of the same filter slices with different antibodies were routinely carried out when the molecular weights of the different tested proteins overlapped. The immunoreactive bands were densitometrically analyzed by the Bio-Rad Image Lab SoftwareTM 6.0.1. β-actin represented the normalizer. Results are shown according to AL group fixed as the unit norm.

### 2.3. In-Gel Activity Assay for Measuring the Activity of SOD2

The activity of SOD2 was assessed on native polyacrylamide gel (Native PAGE) using the riboflavin–nitroblue tetrazolium method [35,36]. Briefly, 30 μg of Sol homogenate was loaded onto native polyacrylamide gel (10%) without β-mercaptoethanol. After running at 4 °C in 1x Tris-glycine buffer (pH = 8.3), the gel was rinsed in a solution of 0.1% nitroblue tetrazolium (NBT) in 50 mM potassium phosphate buffer (PB) (pH 7.8) for 15 min at room temperature (RT). Then, the gel was immersed in a solution of 28 mM tetramethylethylenediamine (TEMED) and 28 mM riboflavin in 50 mM PB buffer (pH 7.8) for 15 min at RT in the dark. After the treatment, the gel was exposed to the light of an electric bulb (100 W) until achromatic areas, representative of SOD2 activity, became visible in contrast with the uniformly intense blue background. Finally, the gel was transferred to distilled water, stopping the excessive continuation of the reaction. SOD2-containing samples with known SOD2 activity were used for the preparation of a standard calibration curve. The densitometric analysis was performed by the Bio-Rad Image Lab SoftwareTM 6.0.1.

### 2.4. Determination of Relative mtDNA Content

Real-time quantitative polymerase chain reaction (qPCR) experiments with SYBR Green were set up to measure mtDNA content relative to nuclear DNA. In total, 3 ng of total DNA, extracted with commercially available kits, was used as a template. mtDNA and β-actin amplicons, respectively, 83 bp and 85 bp long, were obtained using the primers reported in [33]. qPCR conditions were as in [37].

### 2.5. Analysis of Modified Purines

The incidence of oxidized purines, mainly 8-oxo-deoxyguanosine (OH8dG), located in the D-loop region of mtDNA, was determined using formamidopyrimidine DNA glycosylase (Fpg) (New England Biolabs, Beverly, MA, USA, #M0240L) digestion, following the protocol reported in [37]. Shortly, 5 ng of total Fpg-treated and untreated total DNA were the templates used to obtain a 1000 bp amplicon from the mtDNA D-loop region. Primers were as in [33]. Amplicons were revealed by agarose gel electrophoresis. The analysis of the intensity of the bands was conducted through Image Lab Software (Bio-Rad Laboratories Inc., Hercules, CA, USA). The ratio between Fpg-treated and untreated samples was calculated and represented as percentage of the complement to 100.

### 2.6. Statistical Analysis

Statistical analyses were performed using GraphPad Prism software v10.0.1. Results were analyzed using the parametric one-way ANOVA with Dunnett’s multiple comparison test or the nonparametric Kruskal–Wallis with Dunn’s multiple comparison test. Statistical significance was set at *p* < 0.05. All data represent the results of three independent experiments and are expressed as mean + SEM (Standard Error of Mean). Descriptive statistics, Welch’s *t*-test results, effect sizes, and power considerations for all measured parameters were assessed (see Supplementary Table S1).

## 3. Results

### 3.1. Animal Characteristics

Table 1 reports the animals’ body weights measured at the beginning and at the end of the 6 weeks of intervention, immediately before the animals were sacrificed.

Sol weights, instead, were taken after the sacrifice of the animals and also reported as a ratio of body weight (BW). At the end of six weeks, significant differences were found between body weights post-treatment in CR and CR + RSV rats versus AL animals. Significant differences were found between CR and CR + RSV rats versus the AL group in Sol muscle wet weight. Only in CR animals versus AL rats was the Soleus muscle wet weight/BW (mg/g) significantly different.

### 3.2. Effects on Mitochondrial Biogenesis

**Figure 2:**
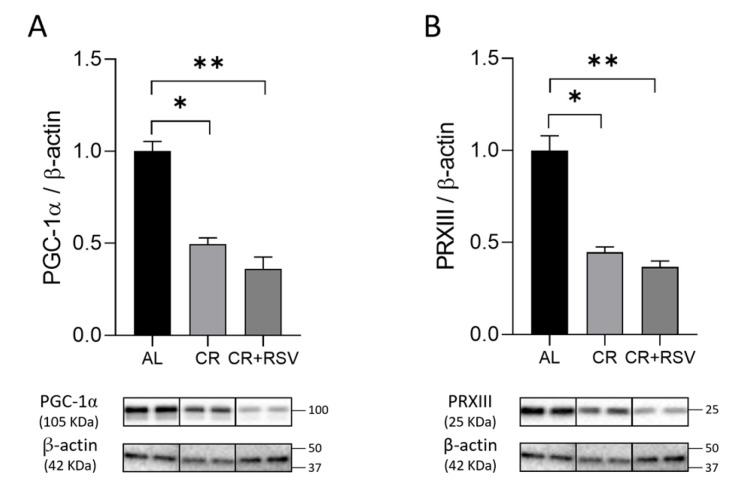
Western blot analysis of PGC-1α (**A**) and PRXIII (**B**) proteins in soleus muscle samples from AL, CR, and CR + RSV rats. The quantification of the intensity of the bands of PGC-1α (**A**) and PRXIII (**B**), normalized to β-actin intensity, is reported above. At the bottom, the immunobands are indicative of two rats from each of the studied groups. Data shown derive from the results of triplicate Western blot experiments analyzed using the Kruskal–Wallis with Dunn’s post test. Bars indicate the mean values + SEM (*n* = 6 per group) for the three experimental groups. Data were normalized against the value of the AL rat group, fixed as 1. Statistical significance: * *p* < 0.05; ** *p* < 0.01; Dunn’s test. AL: ad libitum; CR: caloric restriction; CR + RSV: caloric restriction and resveratrol (50 mg/kg/day; 6 weeks).

Protein expression of the master regulator of mitochondrial biogenesis, peroxisome proliferator-activated receptor gamma coactivator-1α (PGC-1α), and that of peroxiredoxin III (PRXIII), a mitochondrial scavenger of ROS, whose expression is regulated by both mitochondrial biogenesis and ROS presence [38], were evaluated by Western blot experiments in the three groups of rats. Both PGC-1α and PRXIII proteins showed a significant (*p* < 0.05, Dunn’s post test) 50% decrease in the CR group and a significant (*p* < 0.001, Dunn’s post test) 60% decrease in the CR + RSV rats with respect to the AL controls (Figure 2A,B), indicating a marked reduction in mitochondrial biogenesis with both treatments.

### 3.3. Effects on SIRT3-SOD2 Axis

**Figure 3:**
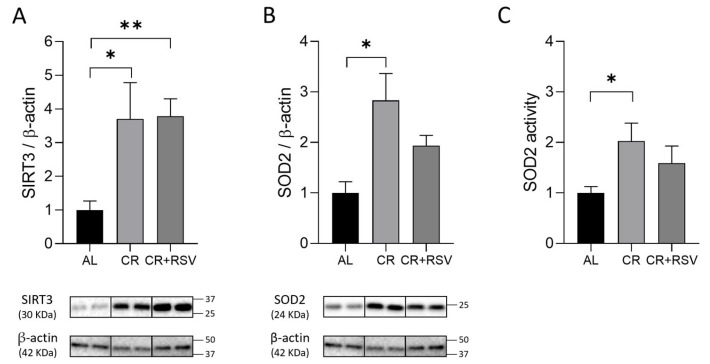
Western blot analysis of SIRT3 (**A**) and SOD2 (**B**) proteins and in-gel activity assay for SOD2 activity (**C**) in soleus muscle samples from AL, CR, and CR + RSV rats. In the histograms (**A**) and (**B**), the quantification of the intensity of the bands of SIRT3 (**A**) and SOD2 (**B**), normalized to β-actin intensity, is reported. At the bottom, the immunobands are indicative of two rats from each of the studied groups. In the histogram (**C**), the quantification of the intensity of achromatic areas representative of SOD2 activity normalized to the SOD2 standard is reported. Data shown derive from the results of triplicate Western blot experiments analyzed using the Kruskal–Wallis with Dunn’s post test. Bars indicate the mean values + SEM (*n* = 6 per group) for the three experimental groups. Data were normalized against the value of the AL rat group, which was fixed as 1. Statistical significance: * *p* < 0.05; ** *p* < 0.01 (Dunn’s test). AL: ad libitum; CR: caloric restriction; CR + RSV: caloric restriction and resveratrol (50 mg/kg/day; 6 weeks).

Since CR was shown to induce the expression of sirtuin 3 (SIRT3) [21], the protein amount of SIRT3 and that of manganese superoxide dismutase 2 (SOD2) were analyzed by Western blot experiments in the three animal groups. The CR diet induced a close to threefold significant (*p* < 0.05, Dunn’s post test) increase in SIRT3 protein amount, and the CR + RSV treatment induced a similar highly significant (*p* < 0.001, Dunn’s test) increase in SIRT3 expression in comparison with the AL group (Figure 3A).

CR also elicited a significant (*p* < 0.05, Dunn’s test), close to twofold, increase in SOD2 protein amount and a significant (*p* < 0.05, Dunn’s test), almost double level of enzyme activity as compared to the AL group (Figure 3B,C). The CR + RSV treatment induced an almost doubled value in SOD2 expression and in SOD2 activity compared with AL (Figure 3B,C), supporting the activation of the SIRT3-SOD2 axis by both ST treatments.

### 3.4. Effects on Mitochondrial Dynamics

**Figure 4:**
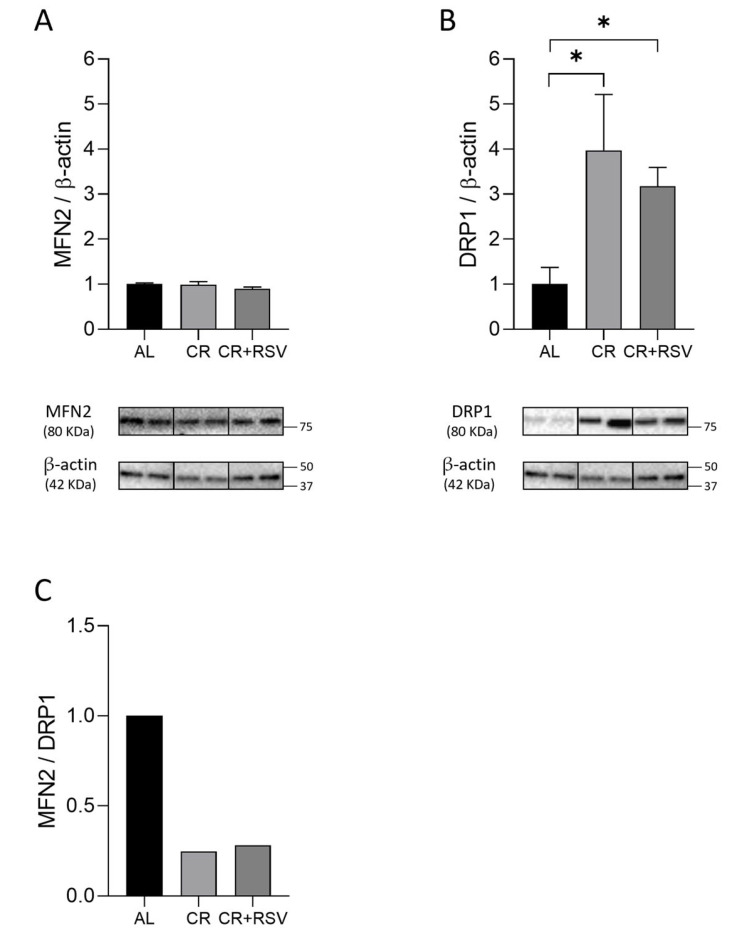
Western blot analysis of MFN2 (**A**) and DRP1 (**B**) proteins and fusion index (**C**) in soleus muscle samples from AL, CR, and CR + RSV rats. The quantification of the intensity of the bands of MFN2 (**A**) and DRP1 (**B**), normalized to β-actin intensity, is reported above. At the bottom, the immunobands are indicative of two rats from each of the studied groups. Data shown derive from the results of triplicate Western blot experiments analyzed using the Kruskal–Wallis with Dunn’s post test. Bars indicate the mean values + SEM (*n* = 6 per group) for the three experimental groups. F.I. (**C**) indicates the ratio between MFN2 and DRP1. Data were normalized against the value of the AL rat group, fixed as 1. Statistical significance: * *p* < 0.05, Dunn’s test. AL: ad libitum; CR: caloric restriction; CR + RSV: caloric restriction and resveratrol (50 mg/kg/day; 6 weeks).

The effects of the treatments on mitochondrial dynamics were assessed through the analysis of the protein amounts of dynamin-related protein 1 (DRP1) and mitofusin 2 (MFN2) by Western blot experiments and the calculation of the fusion index (F.I.) as the MFN2/DRP1 ratio in all rat groups. The protein amounts of fusion protein MFN2 did not show changes across groups (Figure 4A).

The protein amounts of the fission protein DRP1, instead, showed a significant three-fold increase in the CR group versus the AL group (Figure 4B), and a more than two-fold statistically significant increase was found in the CR + RSV group versus AL (*p* < 0.05, Dunn’s test) (Figure 4B). The value of the fusion index (F.I.), determined as the ratio between MFN2/DRP1, presented a marked decrease in both the CR- and CR + RSV-treated groups as compared with the AL group, due to their strong activation of fission (Figure 4C).

### 3.5. Effects on Mitophagy

**Figure 5:**
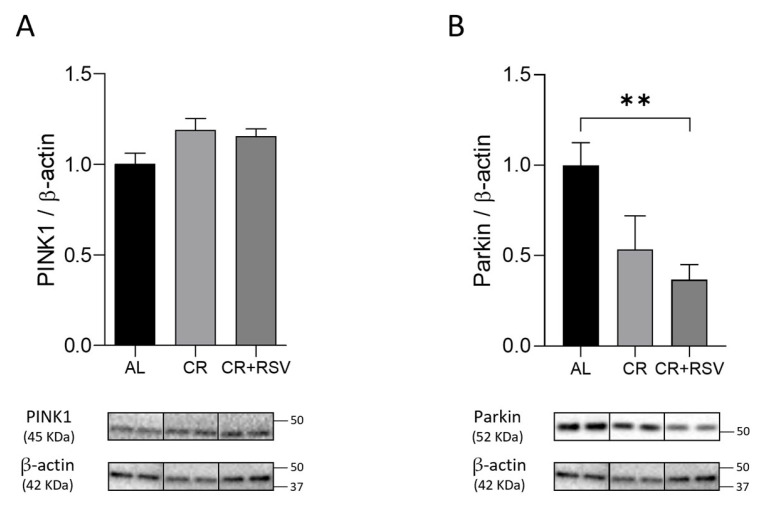
Western blot analysis of PINK1 (**A**) and Parkin (**B**) proteins in soleus muscle samples from AL, CR, and CR + RSV rats. The quantification of the intensity of the bands of PINK1 (**A**) and Parkin (**B**), normalized to β-actin intensity, is reported above. At the bottom, the immunobands are indicative of two rats from each of the studied groups. Data shown derive from the results of triplicate Western blot experiments analyzed using the Kruskal–Wallis with Dunn’s post test. Bars indicate the mean values + SEM (*n* = 6 per group) for the three experimental groups. Data were normalized against the value of the AL rat group, fixed as 1. Statistical significance: ** *p* < 0.01, Dunn’s test. AL: ad libitum; CR: caloric restriction; CR + RSV: caloric restriction and resveratrol (50 mg/kg/day; 6 weeks).

Protein amounts of mediators of the Parkin-dependent mitophagy pathway were also determined. Protein amounts of phosphatase and tensin homolog (PTEN)-induced Kinase 1 (PINK1) and of the E3 ubiquitin–protein ligase Parkin (Parkin) were measured by Western blot experiments in the three groups of rats. Results revealed no significant differences among the groups for PINK1 protein expression (Figure 5A), whereas CR induced a non-significant and CR + RSV a highly significant (*p* < 0.001, Dunn’s test) decrease (−60%) in comparison with the AL group in Parkin amount (Figure 5B).

### 3.6. Effects on mtDNA Maintenance

**Figure 6:**
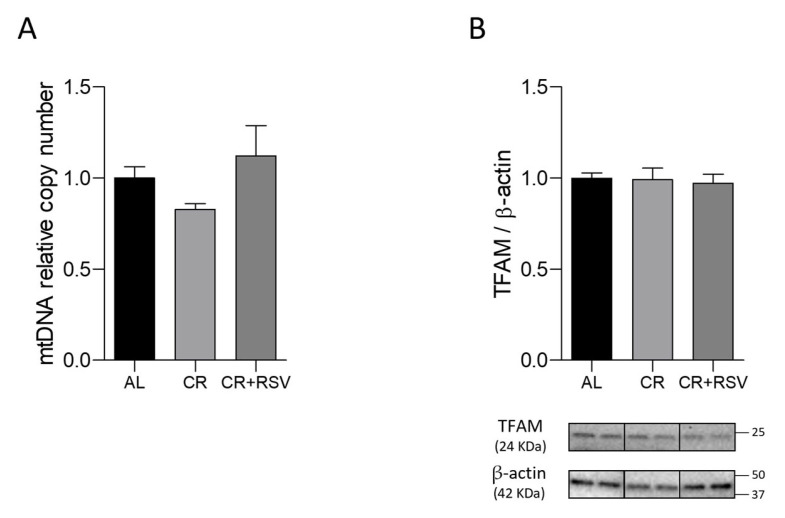
MtDNA relative copy number (**A**) and Western blot analysis of TFAM protein (**B**) in soleus muscle samples from AL, CR, and CR + RSV rats. The quantification of the intensity of the bands of TFAM (**B**), normalized to β-actin intensity, is reported above. At the bottom, the immunobands are indicative of two rats from each of the studied groups. Data shown derive from the results of triplicate Western blot experiments analyzed using the Kruskal–Wallis with Dunn’s post test. Bars indicate the mean values + SEM (*n* = 6 per group) for the three experimental groups. Data were normalized against the value of the AL rat group, fixed as 1. AL: ad libitum; CR: caloric restriction; CR + RSV: caloric restriction and resveratrol (50 mg/kg/day; 6 weeks).

MtDNA maintenance was assessed through determination of the relative content of mtDNA by q-PCR analysis in all groups of rats, and the results, reported in Figure 6A, demonstrated the absence of any significant difference among the groups.

**Figure 7:**
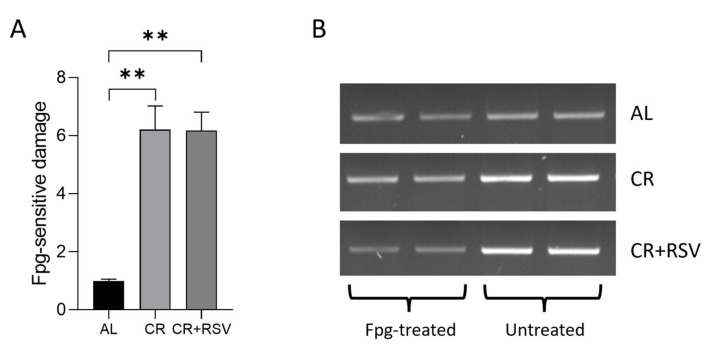
Oxidative damage to mtDNA in soleus muscle samples from AL, CR, and CR + RSV rats. (**A**) Data are means + SEM (*n* = 6 per group) and were normalized against the value of the AL rats, fixed as 1. Bars in the graph represent the ratio between treated and untreated band intensities. Statistical significance: ** *p* < 0.01 (Kruskal–Wallis with Dunn’s post test). (**B**) An agarose gel representing amplicons derived from Fpg-treated and untreated total DNA of two rats from each of the studied groups. AL: ad libitum; CR: caloric restriction; CR + RSV: caloric restriction and resveratrol (50 mg/kg/day; 6 weeks).

The lack of effects of both nutritional treatments on mtDNA copy number was consistent with the unvaried amount of the histone-like protein of mtDNA that is mitochondrial transcription factor A (TFAM) in all groups of animals (Figure 6B), verified by Western blot experiments. The level of oxidative damage was assessed through the determination of the frequency of oxidatively modified bases, mainly 8-hydroxydeoxyguanosine (OH8dG), in the control region of the mtDNA, spanning over the displacement loop (D-loop), which is crucial for mtDNA replication and transcription [38]. The results of such analysis showed a marked five-fold, significant increase in oxidative damage levels in both CR and CR + RSV groups in comparison with the age-matched AL counterpart (Figure 7A,B).

## 4. Discussion

Aging affects skeletal muscle, leading to the progressive loss of mass and strength [39], which severely impacts an individual’s health through an increased incidence of falls, disability, and all-cause mortality. Since such loss is also due to the accumulation of dysfunctional mitochondria [40], one of the main objectives of ongoing research on aging is the identification of treatments, whether nutritional, pharmacological, or lifestyle-based, which may prevent/delay the age-related loss of skeletal muscle [41,42,43], allowing the preservation/regeneration of functional mitochondria. Another relevant goal is the possibility that such treatments may be administered from old age on, thus constituting short-term (ST) regimens, with easy compliance by patients. Moderate CR and administration of RSV are among the treatments more widely used for these purposes [44]. In the present study, the efficacy of ST-CR or combined ST-CR + RSV treatments on mitochondrial biogenesis, mitophagy, and the SIRT3 pathway in Sol from old rats was evaluated. These results have been compared with those of Sol from old rats treated for a short time with RSV [33] (Table 2).

### 4.1. Effects of ST-CR and ST-CR + RSV on Mitochondrial Biogenesis

In Sol from the old rats, the age-related decline in mitochondrial biogenesis was previously demonstrated through the decrease in PGC-1α protein amount [45]. Long-term CR (LT-CR) was demonstrated to fully prevent the age-related reduction of PGC-1α mRNA in the comparison between mixed gastrocnemius muscle from old CR rats (35-month-old) versus that from the AL counterpart (30-month-old), but such effect was not present anymore in the older senescent animals, featuring the same PGC-1α mRNA value in spite of the diet (senescent CR, 40-month-old, versus senescent AL, 35-month-old) [46]. The relevance of the duration of the treatment for skeletal muscle responses was further demonstrated by a study comparing the effects induced by ST-CR (2-week or 2-month) or medium-term (6-month) CR [47]. Results from proton leak kinetics and other analyses indicated that the mechanisms of adaptation to CR were different between short- and medium-term treatments [47]. A more recent paper reported that ST-CR (one-month treatment in 24-month-old rats) in the vastus lateralis skeletal muscle led to a decrease in PGC-1α mRNA in comparison with the age-matched AL counterpart [28]. These latter results were consistent with the present ones, showing a significant decrease in PGC-1α protein amount in the oxidative Sol muscle from both CR and CR + RSV groups in comparison with the age-matched AL counterpart. Also, the previous paper [33] reported a similar significant decrease in PGC-1α protein with ST-RSV treatment, suggesting that all ST treatments tested in old rats were not efficacious in counteracting the age-related decline of mitochondrial biogenesis in the oxidative Sol. Furthermore, the paper comparing the effects of three ST interventions, analogous to those of the present study and to that of the previous one [34], in white gastrocnemius (WG) and red gastrocnemius (RG) also described a positive response to ST-CR as for PGC-1α protein only in the glycolytic WG, but not in the mixed muscle RG, and there was no change at all in either WG or RG with ST-CR + RSV, thus supporting differential responses to the ST treatments depending on the fiber type of the assessed muscle [34]. Differential responses, also to LT-CR, for mitochondrial morphology and dynamics in Sol vs. WG were recently reported and further supported the fiber type-specific sensitivity to the nutritional intervention [14]. Another protein assayed to test mitochondrial biogenesis response in this study was PRXIII, whose amount showed a significant decrease with both ST-CR and ST-CR + RSV. Such a decrease may be explained by the positive correlation identified between PGC-1α and PRXIII in ST-RSV-treated Sol from old rats [33] and may lead to the suggestion that overall mitochondrial biogenesis was further reduced by all ST-treatment with respect to that in untreated AL rats. The effects induced by ST-CR and ST-RSV were not additive, as evident in the ST-CR + RSV group, and suggested that CR and RSV at the concentration (50 mg/kg/day) used in the present study and in the previous one [33] exerted the maximal inhibitory action on mitochondrial biogenesis induction. ST-CR alone obtained the same effect as ST-RSV alone [33] or ST-CR + RSV, likely because both treatments worked, at least partially, through the same pathway. Only one concentration of RSV was used in this study to allow the comparison between the present results and those from [33], as well as those from [34], focused on the effects induced by the same treatments in WG and RG. Therefore, as for the oxidative fibers of Sol, the present results demonstrated the absence of induction of mitochondrial biogenesis with both ST regimens.

### 4.2. Effects of ST-CR and ST-CR + RSV on SIRT3-SOD2 Axis and Antioxidant Proteins

PRXIII expression is controlled not only by the mitochondrial biogenesis pathway, because it is the major antioxidant enzyme active in mitochondria to scavenge H_2_O_2_ produced in the matrix [48,49], thus effectively inhibiting oxidative stress and apoptosis and attenuating cellular damage [50]. PRXIII protein amount was shown to be reduced by the age-related oxidative stress in rat liver [38] and by the oxidative stress originated by mice intestinal ischemia/reperfusion (I/R) injury, as well as by the in vitro simulation of I/R through hypoxia/reoxygenation (H/R) of Caco-2 cells [51]. The latter study also demonstrated that the level of acetylated PRXIII was clearly increased both in vivo and in vitro oxidative stress models and that such an increase impaired the antioxidative action of PRXIII, establishing a functional link with the deacetylase activity of SIRT3 [51]. The described PRXIII activation, induced by SIRT3 deacetylation, drew attention in the present study to assess the expression of SIRT3 deacetylase rather than that of SIRT1. In fact, ST-RSV was shown not to elicit any effect on SIRT1 expression in Sol [33], similarly to what was found in the mixed muscle RG [34]. Mammalian tissue expression and/or activity of mitochondrial sirtuins (SIRT3-5) has been reported to decline with age, as the age-reduced availability of NAD^+^ may affect the NAD^+^-dependent mitochondrial SIRT3-5 enzymes [9]. Moreover, increased ROS production, due to mitochondrial dysfunction, may lead to a damaging post-translational modification of irreversible protein oxidation, i.e., carbonylation of SIRT3 at the level of Cys280. This carbonylation has been shown to induce conformational changes in the SIRT3 active site, thus inhibiting SIRT3 activity and impairing its ability to deacetylate and regulate antioxidant enzyme activity [52]. Several reports supported the existence of a SIRT3-SOD2 axis leading to the activation of SOD2 through its SIRT3-mediated deacetylation [53]. In the literature, CR was demonstrated to activate a defense program to reduce oxidative stress through the increase in SIRT3 expression, activating the mitochondrial antioxidant enzyme SOD2 in in vitro and in vivo studies [21]. In the present work, ST-CR and ST-CR + RSV induced a significant increase in SIRT3 protein amount in comparison with the AL counterpart. Furthermore, expression and activity of SOD2 in both groups of treated old rats were determined. The present results showed that ST-CR induced SOD2 expression and activity, whereas ST-CR + RSV treatment only evoked a tendency to increased expression and activity, thus indicating a partial counteraction by RSV of the responses induced by CR. Effectively, with ST-RSV alone [33], there was no statistical change in SOD2 protein, similar to what was reported in RG [34], whereas only ST-CR induced a statistically significant increase in SOD2 protein in Sol. The increase induced by ST-CR + RSV was not statistically significant, and it might be attributed to a lowering effect by RSV on the maximal effect induced by ST-CR, as with SOD2 expression. Furthermore, these results were supported by those of SOD2 activity, exactly mirroring those of protein expression. Therefore, ST-CR, and to a lesser extent, ST-CR + RSV, triggered the SIRT3-SOD2 axis signaling in oxidative Sol from old rats as a fast/early response. In particular, it might be hypothesized that ST-CR elicited the maximal response as for the SIRT3-SOD2 axis, which was reduced by the combined ST-CR + RSV and abolished SOD2 expression by ST-RSV. These effects might be understandable if CR and RSV pathways partially overlapped when using this RSV concentration, and with RSV prevailing above the CR-mediated SOD2 induction.

### 4.3. Effects of ST-CR and ST-CR + RSV on Mitochondrial Dynamics

Results from the present study indicated that, in the oxidative Sol, ST-CR and ST-CR + RSV did not affect the MFN2 protein amount, namely, fusion was not promoted. On the contrary, a significant increase in MFN2 protein amount, supporting the stimulation of mitochondrial fusion, was shown to be induced by LT-CR, thus likely diluting the damage of dysfunctional organelles [14]. As for mitochondrial fission, the present results showed a significant increase in DRP1 protein amount in ST-CR- and ST-CR + RSV-treated animals. These results are in line with those by [14], where a statistically significant increase in DRP1 protein was reported under LT-CR. Such discrepancies may be explained, at least in part, by the different duration of treatments (ST-CR and ST-CR + RSV vs. LT-CR). According to present results, ST-CR, alone or in combination with RSV, induced a maximal response in fission (more than a two-fold significant increase in comparison with AL values), much higher than that induced by ST-RSV alone (a 0.5-fold increase in comparison with AL values [33]), supporting the hypothesis presented above that CR and RSV pathways partially overlapped, with ST-RSV reducing the maximal ST-CR-induced response, when using this RSV concentration. Present findings supported the diet effect in ST treatments limited to fission, pursuing segregation of dysfunctional mitochondria to likely eliminate them by autophagy [54]. Fusion appeared not sensitive to ST-CR or ST-CR + RSV, as it was not a fast responsive function. These effects led to a marked decrease in the fusion index (F.I.) in both groups of treated rats with respect to the value of age-matched AL rats.

### 4.4. Effects of ST-CR and ST-CR + RSV on Mitophagy

Having demonstrated the ST-mediated induction of fission, leading to elimination of dysfunctional mitochondria, the next step was the analysis of mitophagy signaling by determination of the protein amounts of PINK1 and Parkin [55]. In the present study, a significant decrease in Parkin was found for ST-CR + RSV but not for ST-CR. Parkin appeared more sensitive than PINK1 to nutritional interventions, as described by [7]. Such a beneficial effect of ST-CR + RSV treatment on mitophagy was in line with the enhanced fission-mediated elimination of mitochondria also induced by ST-CR + RSV and ST-CR. Indeed, the previous literature reported the age-related increase in Parkin and PINK1 in mouse tibialis anterior skeletal muscle as a sign of the disruption of mitophagy, leading to the accumulation of dysfunctional mitochondria inside the cells [56]. Like in the present results, LT-CR (18-month CR) induced a significant decrease in Parkin, indicating functional mitophagy and prevention of age-related effects in RG skeletal muscle [13]. According to several studies, aging implies impaired mitophagy, whereas CR triggers mitophagy [57,58,59,60]. Furthermore, Parkin was also indicated to regulate mitochondrial biogenesis and dynamics [61]. In fact, ST-CR + RSV and likely also ST-CR treatment, although at a different rate, induced the elimination of dysfunctional mitochondria through increased fission and rescued mitophagy.

### 4.5. Effects of ST-CR and ST-CR + RSV on mtDNA Maintenance

To widen the analysis of ST nutritional regimens’ effects on mitochondrial quality controls, mtDNA maintenance was assessed through analysis of mtDNA copy number, level of oxidative damage, and TFAM protein amount. Previous findings demonstrated the age-related decrease in mtDNA content in rat Sol, which was counteracted by LT-CR [62]. The histone-like protein of mtDNA, which is TFAM, also presented an age-related decrease in Sol, which was prevented by LT-CR [62]. Present results indicated that ST-CR and ST-CR + RSV did not induce any change in mtDNA content and TFAM amount, further demonstrating the inefficacy of both ST treatments to restore overall mitochondrial biogenesis. Similar results were also obtained by ST-RSV alone [33], suggesting that the 6-week duration of the treatments was too short to fully counteract the age-related mitochondrial dysfunction in Sol from old rats. As for the level of mtDNA oxidative damage, both ST treatments led to a marked and significant increase in comparison with AL rats, as was the case after the treatment with RSV alone [33]. In that same paper [33], an inverse correlation was shown, respectively, between PGC-1α amount and Fpg-sensitive damage and between PRXIII amount and Fpg-sensitive damage, suggestive of the association between decreased mitochondrial biogenesis and increased frequency of oxidatively modified bases in the ST-RSV-treated rats. Present results showed a similar 50% decrease in mitochondrial biogenesis in ST-CR and in ST-CR + RSV in Sol, which could be associated with the higher than five-fold increase in frequency of oxidatively modified bases. Furthermore, although with methionine restriction, closely resembling CR, a tissue-specific reduction in the expression of total BER repair enzymes was shown in the liver [63]. A previous study also reported different tissue-specific changes in the activities of mitochondrial BER enzymes, especially of OGG1, the enzyme responsible for the mtDNA repair of oxidized guanine OH8dG, with CR [64]. Therefore, the present hypothesis is that ST-CR and ST-CR + RSV led to a maximal decrease in OGG1 activity and a sequential increase in frequency of oxidatively modified bases, much higher than the ST-RSV counterparts. One can speculate that the ST treatments impinged on the ROS-mediated retrograde communication from mitochondria to the nucleus [65], preventing the activation of responses to counteract the oxidative stress damages. Furthermore, OGG1 was shown to increase its activity through a SIRT3-mediated deacetylation and to decline during aging in skeletal muscle from 20-month-old mice [66], likely because of the age-related loss of SIRT3 activity/expression. It can be envisioned that recovery of the SIRT3-SOD2 axis by the ST treatments was a sort of fast/early response, different from those involved in other pathways. In addition to this, mitochondrial sirtuins are linked to both opposite arms of ROS activity: regulation of ROS-mediated signaling vs. detoxification of damaging ROS [9]. In fact, mitochondrial sirtuins limit DNA damage by balancing ROS levels through SIRT3, and the SIRT3-SOD2 axis is also involved in protecting cells against genomic instability. According to the present results, the recovery of the SIRT3-SOD2 axis, obtained by ST-CR and ST-CR + RSV, was not shared by other pathways, also involving SIRT3 and affected by aging, as mitochondrial biogenesis and mtDNA maintenance. It was previously shown that SIRT3, the major mitochondrial deacetylase, mediates some of the health benefits of CR in retarding aging, enhancing the activity of enzymes part of the antioxidant network, such as SOD2 [67]. It may be suggested that the ST treatments administered to old rats in the present study induced oxidative Sol muscle responses, fostering the SIRT3-mediated detoxification of damaging ROS, e.g., through an increase in expression/activity of SOD2. At the same time, this action abolished the ROS-mediated signaling relevant for retrograde communication and retrieval of mitochondrial biogenesis, mtDNA repair, and more [68]. It may also be suggested that ST-CR and ST-CR + RSV regained Parkin-dependent mitophagy and promoted fission, with both cooperating in the elimination of dysfunctional mitochondria and in the following reduction of ROS overproduction.

## 5. Limitations

One limitation of the current study is that, due to low tissue availability from the tiny soleus skeletal muscle, we were able to assess only a limited number of mitochondrial markers from each of the tested pathways. Furthermore, the size of the tested groups was small because of compliance with laws requiring the utilization of the minimal number of animals to combine the reliability of the results and the avoidance of useless animal suffering. A second limitation deals with the inclusion of only male rats in the study. In fact, a sex influence on CR effects has been recently reported in the literature, e.g., [69], but the present experimental design included only male rats to make results comparable with those from the previous study on ST-RSV treatment [33] and those from the study on different parts of gastrocnemius [34]. Several different and relevant pathways could not be investigated in the present study, but future research will certainly address the effects elicited by these nutritional treatments in Parkin-independent mitophagy and autophagy; the complex interplay between the mechanistic target of rapamycin (mTOR), the stimulator of muscle protein synthesis, and the AMP-activated protein kinase (AMPK); and the inhibitor of mTOR, which promotes muscle catabolism, as well as in other metabolic pathways controlling muscle homeostasis and performance.

## 6. Conclusions

The present study evaluated a short-term (6 weeks) diet with CR (ST-CR) or combined CR + RSV (ST-CR + RSV) treatments for efficacy in the retrieval of mitochondrial functions affected by aging in the oxidative skeletal soleus muscle (Sol) from 27-month-old rats. Both PGC-1α and PRXIII proteins showed significant marked decreases in the ST-CR and in the ST-CR + RSV rats, suggestive of a further enhancement of the age-related decrease in mitochondrial biogenesis with both treatments. Both ST-CR and ST-CR + RSV led to an increase in SIRT3 protein amount, demonstrating a SIRT3-relevant induction, counteracting the age-related decline of mitochondrial sirtuins. The SIRT3-SOD2 axis was assessed, finding that ST-CR and ST-CR + RSV elicited a significant increase in SOD2 protein amount and activity, although the increase induced by the ST-CR + RSV treatment had a smaller size, suggestive of a partial counteraction by RSV of the responses induced by CR. Therefore, ST-CR and ST-CR + RSV drove recovery of the SIRT3-SOD2 axis as a fast/early response, different from those of other pathways, also involving SIRT3 and affected by aging. As for mitochondrial dynamics, ST-CR and ST-CR + RSV did not affect the MFN2 protein amount, indicative of fusion activities. Differently, the DRP1 protein amount, indicative of fission activities, showed a relevant increase in both the CR and the CR + RSV groups, likely pursuing segregation of dysfunctional mitochondria to eliminate them by autophagy mechanisms. The age-related impairment of mitophagy was counteracted by the ST-CR + RSV- and the ST-CR-induced decrease in Parkin protein amount, leading to the elimination of dysfunctional mitochondria. Such a response enhanced the fission-mediated elimination of mitochondria, also induced by both ST regimens. Neither ST treatment counteracted the reported age-related decrease in mtDNA copy number and TFAM protein amount, further supporting their absence of positive effects on mitochondrial biogenesis. As for the level of mtDNA oxidative damage, both ST treatments led to a marked and significant increase, which might be explained by the supposition that the ST treatments impinged on the ROS-mediated retrograde communication from mitochondria to the nucleus, preventing the activation of responses to counteract the oxidative stress damage. All the effects elicited by ST-CR and ST-CR + RSV in Sol from old rats were specific to the oxidative type fibers and should be assessed also in glycolytic skeletal muscles for a comprehensive understanding of diet effects on skeletal muscle.

## Figures and Tables

**Table 1:** Weights of animals and soleus muscle.

	AL (*n* = 6)	CR (*n* = 6)	CR + RSV (*n* = 6)
Body weight (pre) (g)	570 ± 7.8	562 ± 11.1	568 ± 7.7
Body weight (post) (g)	589 ± 8.3	533 ± 10.0 *	528 ± 8.1 *
Soleus muscle wet weight (g)	0.16 ± 0.004	0.18 ± 0.004 ***	0.17 ± 0.003 ***
Soleus muscle wet weight/BW (mg/g)	0.28 ± 0.005	0.33 ± 0.008 ***	0.31 ± 0.009

AL: ad libitum; CR: caloric restriction; CR + RSV: caloric restriction and resveratrol (50 mg/kg/day; 6 weeks). * *p* < 0.05; *** *p* < 0.001 (Dunnett’s post test vs. AL group); *n* = number of animals analyzed.

**Table 2:** Comparison of results from the previous ST-RSV study and the present findings.

	ST-RSV (Di Lorenzo et al. 2024 [33])	ST-CR (Present Work)	ST-CR + RSV (Present Work)
PGC-1α	−0.5 × fold ***	−0.5 × fold *	−0.6 × fold **
PRXIII	−0.5 × fold ***	−0.5 × fold *	−0.6 × fold **
SIRT1	=	N.D.	N.D.
SIRT3	N.D.	+2.5 × fold *	+2.5 × fold **
TFAM	=	=	=
SOD2	=	+1.8 × fold *	+0.8 × fold
SOD2 activity	N.D.	+1.0 × fold *	+0.5 × fold
GSH/GSSG	+0.2 × fold **	N.D.	N.D.
MtDNA copy number	=	=	=
Fpg-sensitive damage	+0.5 × fold *	+5 × fold **	+5 × fold **
MFN2	=	=	=
DRP1	+0.5 × fold *	+3.0 × fold *	+2.0 × fold *
PINK1	N.D.	=	=
Parkin	N.D.	−0.5 × fold	−0.6 × fold **

ST-RSV: short-term resveratrol (50 mg/kg/day; 6 weeks); ST-CR: short-term caloric restriction; ST-CR + RSV: short-term caloric restriction and resveratrol (50 mg/kg/day; 6 weeks); N.D.: not determined; =: no change versus ad libitum; * *p* < 0.05 versus ad libitum; ** *p* < 0.01 versus ad libitum; *** *p* < 0.001 versus ad libitum.

## Data Availability

The original contributions presented in this study are included in the article. Further inquiries can be directed to the first, last, or corresponding authors.

## Supplementary Materials

The following supporting information can be downloaded at https://www.mdpi.com/article/10.3390/antiox14091125/s1. Figure S1–S12: Original Western blot image; Table S1: Descriptive statistics, Welch’s *t*-test results, effect sizes, and power considerations for all measured parameters.
