# Supplementary material for: Recovery of SIRT3-SOD2 Axis and Mitophagy by Short-Term Calorie Restriction in Old Rat Soleus Skeletal Muscle
Source: Antioxidants (Basel). 2025 Sep 17;14(9):1125. doi: 10.3390/antiox14091125 (PMC12466821; doi:10.3390/antiox14091125)
Supplement: Supplementary file 1 [file antioxidants-14-01125-s001.zip › antioxidants-3762645-supplementary.pdf]

ORIGINAL BLOTS

SUPPLEMENTARY MATERIALS AND METHODS

Immunoblot and antibodies

Preliminary titration experiments allowed us to establish the amounts of protein extracts which gave a signal in the linear range of the relation: densitometric value/blotted proteins. Different exposures of each western blot, in the time range between 1 s and 5 min, were taken to ensure the linearity of the response for all assayed proteins. The densitometric value of the optical density (OD) units of each protein band was then related to the OD units number of the respective band of β-actin (in the corresponding lane).

Before to perform the protein quantification experiments each antibody was tested separately verifying the presence of a single immunoreactive band of the expected molecular weight for all of them. Having verified the migration of all the immunoreacted bands the filter was cut in various slices after the transfer, having care to include a space of at least 1 cm above and 1 cm below the expected band. In this way the same filter could be probed with different antibodies.

Reprobing a western blot saves time and conserves sample while allowing optimization to be performed as needed. The stripping buffer (Restore Stripping Buffer, ThermoFisher Scientific, Waltham, Massachusetts, USA #21059) is used to dissociate and strip primary and secondary antibodies from western blots so that membranes can be reprobed under alternate conditions or with another antibody to detect a different protein target.

In the western blotting experiments for the present study, stripping and reprobing of the same filter slices with different antibodies were routinely carried-out when the molecular weights of different tested proteins were the same and overlapped.

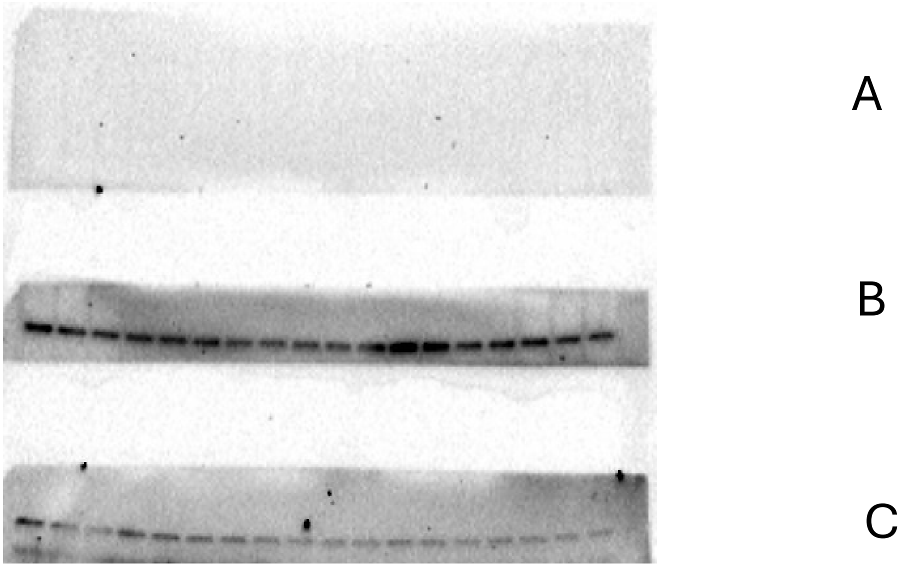

Supplementary Figure Reprobing. Representative western blotting reprobing for proteins in rat soleus skeletal muscle. Representative reprobing of blots of proteins used in the manuscript.
A) Superior slice: stripped but not rehybridized (testing the efficacy of stripping)
B) Intermediate slice: stripped and rehybridized for β-actin protein
C) Inferior slice: stripped and rehybridized for TFAM protein

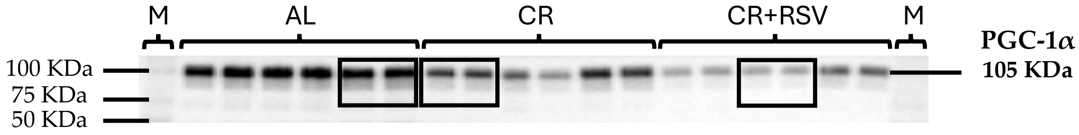

Supplementary Figure S1. Representative western blotting for PGC-1α in rat soleus skeletal muscle. Representative whole blots of PGC-1α protein used in manuscript.
M (Marker of molecular weight), AL (Ad Libitum, controls), CR (Caloric Restriction), CR+RSV (Caloric Restriction plus Resveratrol administration, 50 mg/kg/day). The bands enclosed in the boxes are reported in Figure 1A.

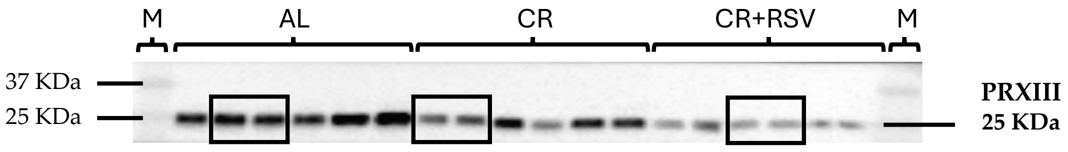

Supplementary Figure S2. Representative western blotting for PRXIII in rat soleus skeletal muscle. Representative whole blots of PRXIII protein used in manuscript.
M (Marker of molecular weight), AL (Ad Libitum, controls), CR (Caloric Restriction), CR+RSV (Caloric Restriction plus Resveratrol administration, 50 mg/kg/day). The bands enclosed in the boxes are reported in Figure 1B.

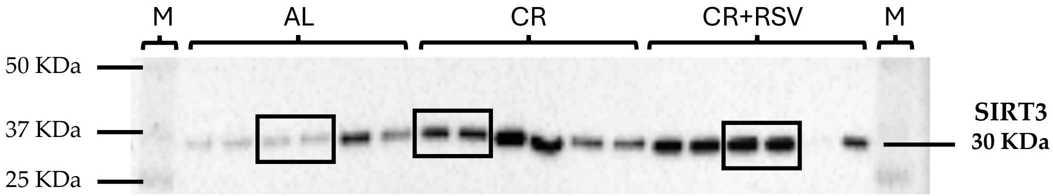

Supplementary Figure S3. Representative western blotting for SIRT3 in rat soleus skeletal muscle. Representative whole blots of SIRT3 protein used in manuscript.
M (Marker of molecular weight), AL (Ad Libitum, controls), CR (Caloric Restriction), CR+RSV (Caloric Restriction plus Resveratrol administration, 50 mg/kg/day). The bands enclosed in the boxes are reported in Figure 2A.

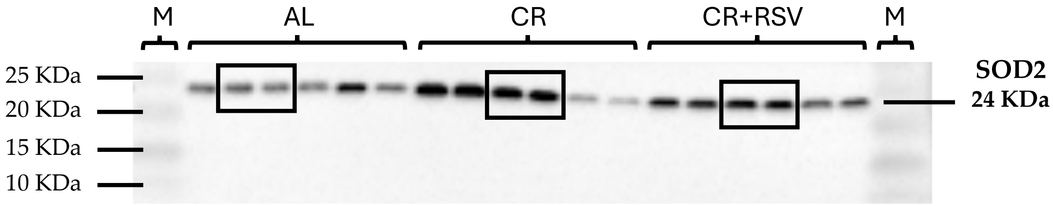

Supplementary Figure S4. Representative western blotting for SOD2 in rat soleus skeletal muscle. Representative whole blots of SOD2 protein used in manuscript.

M (Marker of molecular weight), AL (Ad Libitum, controls), CR (Caloric Restriction), CR+RSV (Caloric Restriction plus Resveratrol administration, 50 mg/kg/day). The bands enclosed in the boxes are reported in Figure 2B.

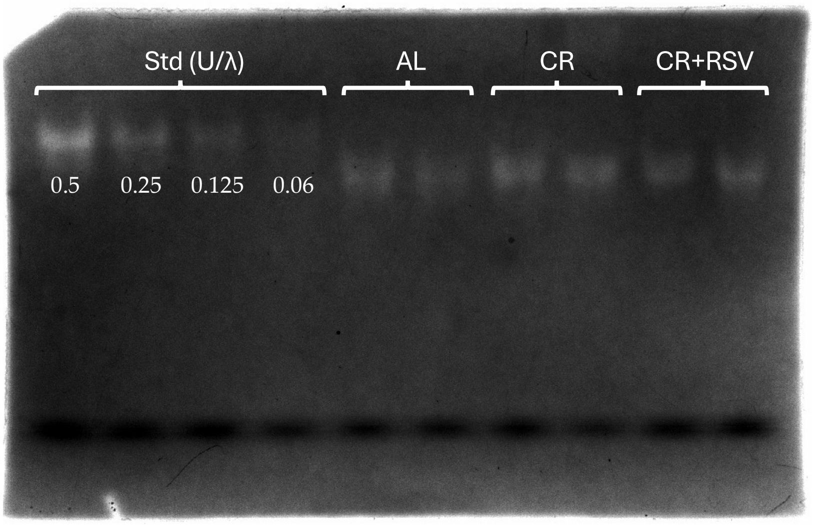

Supplementary Figure S5. Representative In-gel activity assay for measuring SOD2 activity in rat soleus skeletal muscle. Achromatic areas represent SOD2 activity. Known decreasing concentrations of SOD2 are loaded into the gel as standard.

Std (Standard: SOD2 at known concentration), AL (Ad Libitum, controls), CR (Caloric Restriction), CR+RSV (Caloric Restriction plus Resveratrol administration, 50 mg/kg/day). Results of the densitometric analysis are histographically reported in Figure 2C.

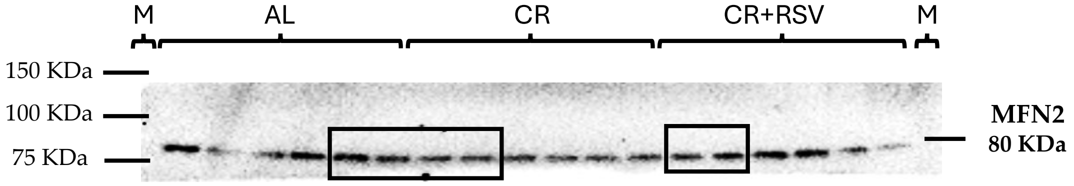

Supplementary Figure S6. Representative western blotting for MFN2 in rat soleus skeletal muscle. Representative whole blots of MFN2 protein used in manuscript.
M (Marker of molecular weight), AL (Ad Libitum, controls), CR (Caloric Restriction), CR+RSV (Caloric Restriction plus Resveratrol administration, 50 mg/kg/day). The bands enclosed in the boxes are reported in Figure 3A.

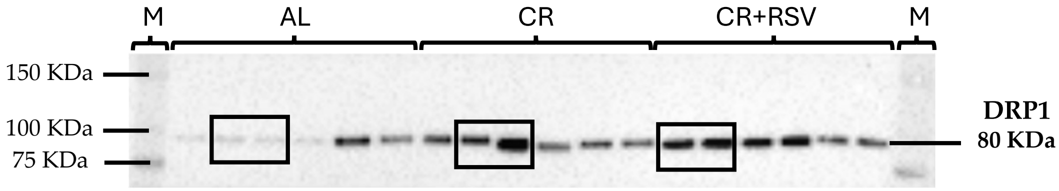

Supplementary Figure S7. Representative western blotting for DRP1 in rat soleus skeletal muscle. Representative whole blots of DRP1 protein used in manuscript.

M (Marker of molecular weight), AL (Ad Libitum, controls), CR (Caloric Restriction), CR+RSV (Caloric Restriction plus Resveratrol administration, 50 mg/kg/day). The bands enclosed in the boxes are reported in Figure 3B.

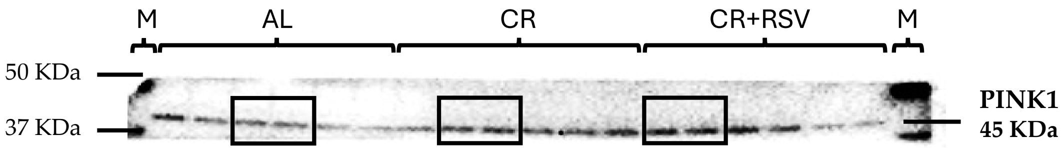

Supplementary Figure S8. Representative western blotting for PINK1 in rat soleus skeletal muscle. Representative whole blots of PINK1 protein used in manuscript.

M (Marker of molecular weight), AL (Ad Libitum, controls), CR (Caloric Restriction), CR+RSV (Caloric Restriction plus Resveratrol administration, 50 mg/kg/day). The bands enclosed in the boxes are reported in Figure 4A.

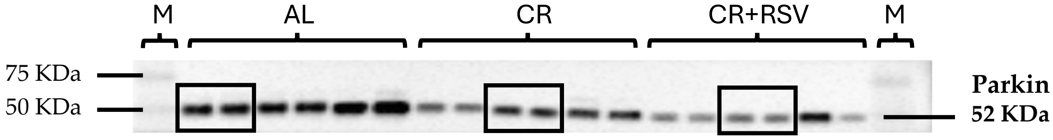

Supplementary Figure S9. Representative western blotting for Parkin in rat soleus skeletal muscle. Representative whole blots of Parkin protein used in manuscript.
M (Marker of molecular weight), AL (Ad Libitum, controls), CR (Caloric Restriction), CR+RSV (Caloric Restriction plus Resveratrol administration, 50 mg/kg/day). The bands enclosed in the boxes are reported in Figure 4B.

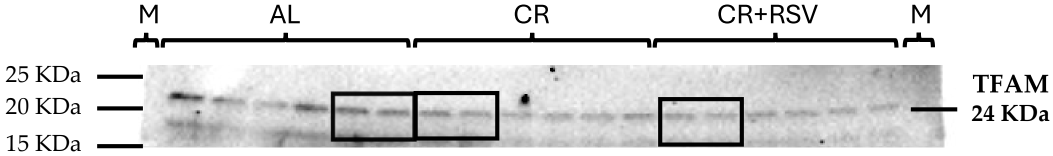

Supplementary Figure S10. Representative western blotting for TFAM in rat soleus skeletal muscle. Representative whole blots of TFAM protein used in manuscript.

M (Marker of molecular weight), AL (Ad Libitum, controls), CR (Caloric Restriction), CR+RSV (Caloric Restriction plus Resveratrol administration, 50 mg/kg/day). The bands enclosed in the boxes are reported in Figure 5B.

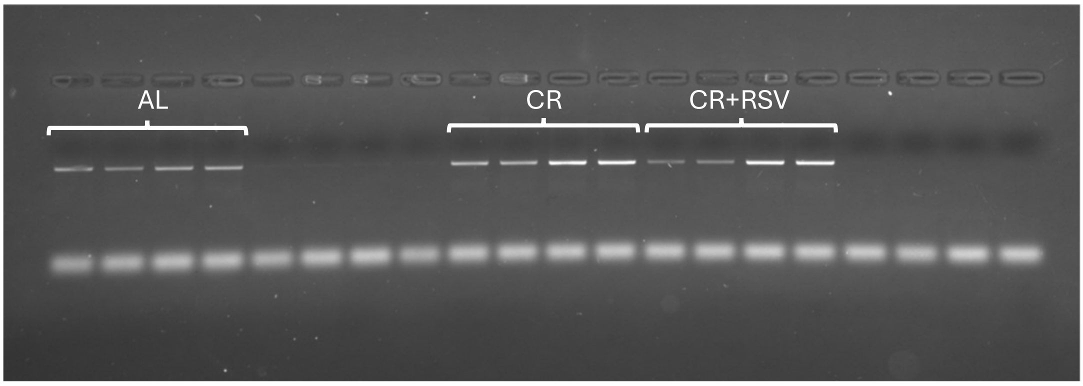

Supplementary Figure S11. Representative agarose gel for amplicons in soleus rat skeletal muscle. Representative agarose gel showing amplicons obtained from Fpg-treated and untreated total DNA.

AL (Ad Libitum, controls), CR (Caloric Restriction), CR+RSV (Caloric Restriction plus Resveratrol administration, 50 mg/kg/day). The bands enclosed in the boxes are reported in Figure 6B.

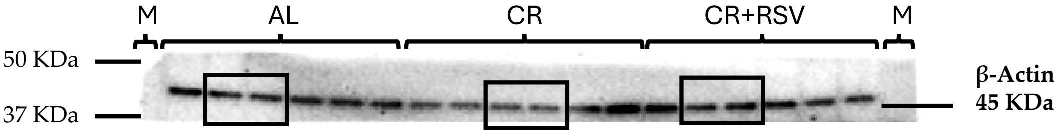

Supplementary Figure S12. Representative western blotting for β-Actin in rat soleus skeletal muscle. Representative whole blots of β-Actin protein used in manuscript.
M (Marker of molecular weight), AL (Ad Libitum, controls), CR (Caloric Restriction), CR+RSV (Caloric Restriction plus Resveratrol administration, 50 mg/kg/day). The bands enclosed in the boxes are reported in Figure 1A-B, 2A-B, 3A-B, 4A-B, 5B.

Supplementary Table 1. *Descriptive statistics, Welch's t-test results, effect sizes, and power considerations for all measured parameters.*

For each marker, group means ± SD are reported for ad libitum (AL), caloric restriction (CR), and CR + resveratrol (CR+RSV) conditions (*n* = 6 per group). Pairwise comparisons (CR vs AL, CR+RSV vs AL, CR+RSV vs CR) include Welch's *t*-test mean differences with 95% confidence intervals and *p*-values, as well as standardized effect sizes (Cohen's *d* and Hedges' *g* with 95% confidence intervals). One-way ANOVA effect sizes (η^2) are also provided. For reference, the minimum detectable effect size for this design is $d \approx 1.62$ ($\alpha = 0.05$, 80% power). Significant results correspond to large standardized effects, while non-significant findings are generally associated with small-to-moderate effects, which may reflect limited power rather than the absence of true biological differences.

Marker	Comparison	Mean diff	95% CI (mean diff)	Welch df	p (Welch)	Cohen d	Hedges g	95% CI (d)	MDE_d (80% power)	eta_squared (ANOVA)
PGC1a	CR vs AL	-0,8501	[-1.09, -0.606]	8,44	0.0000	-4,6	-4,25	[-5.92, -3.28]	1,62	0,85
PGC1a	CR+RSV vs AL	-1,0755	[-1.39, -0.762]	9,71	0.0000	-4,43	-4,09	[-5.72, -3.14]	1,62	0,85
PGC1a	CR+RSV vs CR	-0,2254	[-0.508, 0.0572]	7,61	0.1025	-1,07	-0,99	[-2.41, 0.27]	1,62	0,85
PINK1	CR vs AL	0,1013	[-0.00474, 0.207]	9,98	0.0592	1,23	1,13	[-0.06, 2.52]	1,62	0,29
PINK1	CR+RSV vs AL	0,0823	[-0.00833, 0.173]	8,83	0.0700	1,19	1,1	[-0.12, 2.50]	1,62	0,29
PINK1	CR+RSV vs CR	-0,019	[-0.112, 0.0744]	8,64	0.6548	-0,27	-0,25	[-1.58, 1.05]	1,62	0,29
SOD2 protein	CR vs AL	0,5295	[0.13, 0.929]	6,69	0.0168	1,83	1,69	[0.45, 3.21]	1,62	0,47
SOD2 protein	CR+RSV vs AL	0,2705	[0.076, 0.465]	9,92	0.0113	1,79	1,65	[0.50, 3.08]	1,62	0,47
SOD2 protein	CR+RSV vs CR	-0,259	[-0.657, 0.139]	6,43	0.1646	-0,91	-0,84	[-2.30, 0.49]	1,62	0,47
MFN2	CR vs AL	-0,0143	[-0.205, 0.177]	6,59	0.8631	-0,1	-0,1	[-1.49, 1.28]	1,62	0,15
MFN2	CR+RSV vs AL	-0,1113	[-0.232, 0.00967]	8,78	0.0670	-1,21	-1,11	[-2.52, 0.10]	1,62	0,15
MFN2	CR+RSV vs CR	-0,097	[-0.295, 0.101]	8,16	0.2922	-0,65	-0,6	[-1.98, 0.68]	1,62	0,15
PARKIN	CR vs AL	-0,392	[-0.66, -0.124]	5,87	0.0119	-2,08	-1,92	[-3.50, -0.66]	1,62	0,64
PARKIN	CR+RSV vs AL	-0,5331	[-0.819, -0.247]	8,76	0.0023	-2,44	-2,25	[-3.75, -1.13]	1,62	0,64
PARKIN	CR+RSV vs CR	-0,1411	[-0.324, 0.0414]	6,87	0.1100	-1,06	-0,98	[-2.43, 0.31]	1,62	0,64
PRXIII	CR vs AL	-0,5144	[-0.705, -0.324]	6,29	0.0005	-3,78	-3,49	[-5.18, -2.38]	1,62	0,85
PRXIII	CR+RSV vs AL	-0,5898	[-0.781, -0.399]	6,63	0.0002	-4,26	-3,94	[-5.65, -2.88]	1,62	0,85
PRXIII	CR+RSV vs CR	-0,0754	[-0.166, 0.0148]	9,85	0.0919	-1,08	-1	[-2.37, 0.21]	1,62	0,85
DRP1	CR vs AL	0,2405	[-0.0175, 0.499]	5,89	0.0626	1,32	1,22	[-0.10, 2.74]	1,62	0,34
DRP1	CR+RSV vs AL	0,1764	[0.0759, 0.277]	9,88	0.0029	2,26	2,09	[0.97, 3.55]	1,62	0,34
DRP1	CR+RSV vs CR	-0,0641	[-0.322, 0.194]	6,1	0.5672	-0,35	-0,32	[-1.76, 1.06]	1,62	0,34
SIRT3	CR vs AL	0,2791	[-0.00706, 0.565]	5,62	0.0542	1,4	1,29	[-0.04, 2.84]	1,62	0,4
SIRT3	CR+RSV vs AL	0,2881	[0.149, 0.428]	7,57	0.0016	2,78	2,56	[1.43, 4.12]	1,62	0,4
SIRT3	CR+RSV vs CR	0,009	[-0.282, 0.3]	7,15	0.9439	0,04	0,04	[-1.32, 1.40]	1,62	0,4
TFAM	CR vs AL	-0,0118	[-0.253, 0.23]	7,07	0.9114	-0,07	-0,06	[-1.43, 1.30]	1,62	0,01
TFAM	CR+RSV vs AL	-0,0428	[-0.241, 0.155]	8,03	0.6320	-0,29	-0,27	[-1.62, 1.04]	1,62	0,01
TFAM	CR+RSV vs CR	-0,031	[-0.298, 0.236]	9,55	0.7998	-0,15	-0,14	[-1.45, 1.14]	1,62	0,01
SOD2 activity	CR vs AL	0,2685	[-0.0714, 0.608]	6,18	0.1019	1,11	1,02	[-0.29, 2.51]	1,62	0,17
SOD2 activity	CR+RSV vs AL	0,1537	[-0.172, 0.48]	6,29	0.2953	0,66	0,61	[-0.74, 2.06]	1,62	0,17
SOD2 activity	CR+RSV vs CR	-0,1148	[-0.523, 0.293]	9,98	0.5447	-0,36	-0,33	[-1.65, 0.92]	1,62	0,17
mtDNA	CR vs AL	-1050,0923	[-2.45e+03, 347]	7,61	0.1203	-1,01	-0,93	[-2.35, 0.33]	1,62	0,12
mtDNA	CR+RSV vs AL	740,8195	[-2.91e+03, 4.4e+03]	6,37	0.6411	0,28	0,26	[-1.11, 1.68]	1,62	0,12
mtDNA	CR+RSV vs CR	1790,9118	[-1.85e+03, 5.43e+03]	5,39	0.2668	0,71	0,66	[-0.74, 2.17]	1,62	0,12
Oxidative damage	CR vs AL	39,047592	[20.9, 57.2]	5,03	0.0026	3,18	2,94	[1.70, 4.66]	1,62	0,73
Oxidative damage	CR+RSV vs AL	38,83113	[25.7, 51.9]	5,06	0.0006	4,39	4,05	[2.91, 5.87]	1,62	0,73
Oxidative damage	CR+RSV vs CR	-0,2164614	[-19.9, 19.5]	9,09	0.9807	-0,01	-0,01	[-1.32, 1.29]	1,62	0,73
